# Identifying Genomic Signatures of Positive Selection to Predict Protective Genomic Loci in the Cohort of Lithuanian Clean-Up Workers of the Chornobyl Nuclear Disaster

**DOI:** 10.3390/cimb45040195

**Published:** 2023-04-03

**Authors:** Gabrielė Žukauskaitė, Ingrida Domarkienė, Aušra Matulevičienė, Svetlana Dauengauer-Kirlienė, Vaidutis Kučinskas, Laima Ambrozaitytė

**Affiliations:** Department of Human and Medical Genetics, Institute of Biomedical Sciences, Faculty of Medicine, Vilnius University, Universiteto Str. 3, LT01513 Vilnius, Lithuania; gabriele.zukauskaite@mf.vu.lt (G.Ž.);

**Keywords:** adaptiveness, positive selection, protective genome variation, whole-genome sequencing, RAiSD, Lithuanian clean-up workers of Chornobyl nuclear disaster, selective sweep

## Abstract

Some people resist or recover from health challenges better than others. We studied Lithuanian clean-up workers of the Chornobyl nuclear disaster (LCWC) who worked in the harshest conditions and, despite high ionising radiation doses as well as other factors, continue ageing relatively healthily. Thus, we hypothesised that there might be individual features encoded by the genome which act protectively for better adaptiveness and health that depend on unique positive selection signatures. Whole-genome sequencing was performed for 40 LCWC and a control group composed of 25 men from the general Lithuanian population (LTU). Selective sweep analysis was performed to identify genomic regions which may be under recent positive selection and determine better adaptiveness. Twenty-two autosomal loci with the highest positive selection signature values were identified. Most important, unique loci under positive selection have been identified in the genomes of the LCWC, which may influence the survival and adaptive qualities to extreme conditions, and the disaster itself. Characterising these loci provide a better understanding of the interaction between ongoing microevolutionary processes, multifactorial traits, and diseases. Studying unique groups of disease-resistant individuals could help create new insights for better, more individualised, disease diagnostics and prevention strategies.

## 1. Introduction

Survival and adjustment to the ever-changing environmental conditions are essential components of adaptation and evolution in a population. Adaptation is directly dependent on the forces of natural selection. Therefore, natural selection is a crucial factor in evolution that has the power to alter the gene pool of a population [1]. In this way, past and future populations are formed by natural selection acting on the population’s genetic structure through phenotypes and adaptive properties in the current environment. The genetic structure differs between populations, mainly due to the different microevolutionary processes which result in other characteristics.

The increasing availability of large-scale genomic data has led to many genome-wide association studies being performed, including those on the adaptation of different populations. These studies allowed us to gain insights into new candidate genomic loci, and genes and their interactions regarding the processes of adaptation to different environments, such as nutrition and diseases [2,3]. Identified adaptive genomic loci allow us to clarify how natural selection shapes the variation of the genome in different populations and provides essential knowledge about the influence of these genomic loci on biological functions and phenotypes. For example, it was found that a lower frequency of non-synonymous changes in genes, representing the effect of purifying selection, is directly correlated with the incidence of Mendelian diseases [4]. Positive natural selection was also found to act on genes involved in the molecular mechanisms of cancer [5]. This may be due to the inclusion of critical regulatory mechanisms (e.g., DNA damage repair) in the carcinogenic processes, which may protect against cancer development. In addition, some genomic loci under positive natural selection may potentially influence etiopathogenesis and frequency of multifactorial diseases, such as cardiovascular diseases (*MMP3, CYP3A,* and *AGT* genes) [6,7,8], type II diabetes (*CAPN10* gene) [9], and asthma (*IL13, IL4,* and *IL1A* genes) [10,11,12]. These are only a few examples, as more than 2000 genes have already been identified under positive natural selection and are associated with numerous phenotypic traits [13]. Therefore, identifying genomic signatures of positive selection could assist in finding protective genomic loci. These loci could be used as potential targets for gene therapy to create new disease diagnostics, prevention, and treatment strategies, thus moving towards personalised medicine. One of the best-known examples is a protective genome variant in the *CCR5* gene, which disrupts the activity of the encoded receptor. This genome variant prevents HIV from entering the cell, thus protecting the individual from the disease. This knowledge has been used to develop a treatment strategy [14].

Each population has unique genomic characteristics that may lead to different disease susceptibility or other adaptive properties. The Lithuanian population is genetically similar to neighbouring European populations, such as Slavs or Finno-Ugrians [15]. Analysis of Y chromosome haplogroups identified the genetic similarity of the Lithuanian population with Latvians and Estonians [15]. These populations are under similar environmental pressures and natural selection forces; this is one reason for their similar genomic characteristics. However, as populations share similarities between genetic traits, they also differ between some, therefore, it is important to find the genomic uniqueness of each population. To achieve this goal, studies have been conducted by analysing the genetic structure of the Lithuanian ethnolinguistic groups [16], or by examining the genomic loci affected by positive natural selection using traditional statistical analysis methods (e.g., F_ST_, Tajima D) [17]. Results show that Lithuanians are a homogenous population [16,18,19]. Positive natural selection acts on the genes related to eating habits (*PNLIP* and *PPARD*), skin pigmentation (*SLC24A5, TYRP1* and *PPARD*), and immune response (*BRD2, HLA-DOA, IL26* and *IL22*) [18]—36 genomic loci have been identified [17]. These findings prove that positive natural selection shapes traits of the Lithuanian population related to adaptation. New approaches should be added to the traditional statistics (e.g., F_ST_, extended haplotype homozygosity, Tajima D) to complement existing results. Thus, we applied a new analysis method to detect positive natural selection signatures in the Lithuanian subpopulation of Lithuanian clean-up workers of Chornobyl (in this study, the name Chornobyl (transcribed from the Ukrainian language) has been chosen instead of the more widespread name Chernobyl (transcribed from the Russian language)) nuclear power plant disaster (LCWC) using the RAiSD [20] tool. We chose to investigate the specific LCWC group as a different strategy to analyse the unique genetic makeup of a population.

In 1986 in Chornobyl, Ukraine, a nuclear disaster occurred after the failure of a nuclear power plant reactor. About 600,000 people, during the period between 1986–1990, were recruited to clean-up the consequences of the disaster. Subsequently, they experienced long-term effects of ionising radiation (IR) (via foods of plant and animal origin, water, and air) [21]. As a result, the incidence of thyroid cancer increased [22]. A large number of clean-up workers suffered—and suffer to this day—from various diseases, post-traumatic stress disorder, or depression [23]. In addition, high doses of IR increase the risk of cancer, and thyroid and eye diseases [24]. A study of the Estonian clean-up workers of the Chornobyl disaster showed an increased mortality rate compared to the general Estonian population. The causes of increased mortality were cancer of the mouth or pharynx, cancerous diseases associated with increased alcohol consumption, and suicides [25]. In the area of Bryansk (Russia), where high radioactive contamination was also detected after the Chornobyl disaster, the frequency of congenital anomalies (for example, polydactyly) in newborns statistically significantly increased [26]. Other territories and countries in relative proximity to Chornobyl had increased quantities of long half-life (approximately 30 years) radionuclides [27]. Being that the half-life of these radionuclides is calculated in decades, their effects will be felt over several generations.

Massive catastrophes, wars, and ecological disasters serve as quasi-experiments. For this reason, populations that survive become precious objects for studies of adaptation. Studying adaptiveness and survival in such conditions is especially relevant in these times of uncertainty when we live in the shadow of nuclear threat. Potentially, some part of the resistance to the toxic and traumatising environment may depend on the unique genome variation and positive selection signatures. Until now, genomic studies of LCWC were predominantly cytogenetic, identifying significant changes in clean-up workers’ genomic stability [28,29]. This study—to our knowledge, the first of its kind—used LCWC whole-genome sequencing data to evaluate loci under positive selection and their significance to human health and adaptiveness. We performed the analysis in the cohorts of LCWC and the general Lithuanian population (LTU). We contribute new information on positive natural selection signatures in LTU and LCWC cohorts, and possible protective genetic variation, as a candidate for future research and application.

## 2. Materials and Methods

### 2.1. Participants and Samples

LCWC is a unique population group and resource for this study. These individuals not only experienced the consequences of the Chornobyl nuclear power plant disaster itself but also participated in its clean-up. Among the surviving LCWC, there are both diseased individuals and those who are ageing relatively healthily. They survived extreme conditions and adapted to the lifelong effects of ionising radiation (IR) and the consequences of the disaster itself (such as psychological trauma, high alcohol consumption, etc.). Samples were collected between 2019–2021, about 30 years after the Chornobyl nuclear disaster. Whole-genome sequencing (WGS) was performed for 40 men who, according to the questionnaire responses, experienced the harshest conditions (such as higher IR doses, working site, nature of work, time of allocation, period of working time, etc.) during clean-up work in Chornobyl. We compared their whole-genome data with the WGS dataset of 25 men from LTU [30]. The LTU dataset was used as the control for common putative selection sweeps. DNA was extracted from peripheral blood leukocytes using the phenol-chloroform-isoamyl alcohol method according to the laboratory-approved methodology (LCWC samples) or using QIAGEN GENTRA^®^ Puregene^®^ Blood Kit (Qiagen GmbH, Germany) extraction protocol (LTU samples). DNA concentration and purity were determined with a NanoDrop^®^ ND-1000 (Thermo Fisher Scientific, Wilmington, DE, USA) spectrophotometer. All procedures performed in this study adhered to the institutional and national research committee ethical standards. Written informed consent was received from all study participants.

During the sample collection, LCWC were required to complete a questionnaire that allowed the collection of information regarding individuals’ experienced IR dose (according to the expedition documents), clinical data, and more. At the time of the study, the average age of LCWC was 64 years (age variation between 50–78 years). The average age of LTU group individuals was 36 years (age variation between 29–49 years). There was no possibility of assimilating the two cohorts by the average age. This may be considered a limitation of the study. However, the age difference (in one generation) does not affect natural selection significantly. Moreover, the analysis method (see Section 2.2) is designed to analyse recent positive selection (considered within the past 5000–100,000 years) signatures [31].

Lithuanian clean-up workers of the Chornobyl nuclear disaster have experienced higher than usual doses of IR. The average annual dose due to natural sources of IR in Lithuania is 2.2 mSv [32]. The annual effective dose limit recommendation for occupational exposure is up to 50 mSv per year [33]. In this study, doses of less than 100 mSv were observed in 7.5% (3 of 40 individuals) of LCWC, while 100–200 mSv and higher than 200 mSv doses were observed in 37.5% (15 of 40) and 55% (22 of 40) of individuals, respectively.

### 2.2. Whole-Genome Sequencing and Data Analysis

Sequencing and primary quality control of raw data files (.fastq) were performed using the Illumina NovaSeq 6000 (Illumina, San Diego, CA, USA) sequencing system and standard DRAGEN version 3.6.4 workflow (the Centre for Genomics and Transcriptomics (CeGaT), Germany; under the contract with Vilnius University). On average, 94.72% of the reads were mapped to the reference genome hg19. Sequencing was performed at coverage of 26.88–61.38× (an average of 36.27×) per both sample groups. An amount of 100 ng DNA was paired-end sequenced in 2 × 150 bp mode using TruSeq^®^ Nano DNA Library Prep Kit (Illumina Inc., San Diego, CA, USA). Demultiplexing of the sequencing reads was performed with Illumina bcl2fastq (2.20). Adapters were trimmed with Skewer (version 0.2.2). Quality trimming of the reads was not performed. The quality of the .fastq files was analysed with FastQC (version 0.11.5-cegat). Sequencing quality control Q30 values were above 88.59%.

To identify genomic loci under positive selection which are unique to LCWC, RAiSD v2.9 (Raised Accuracy in Sweep Detection) software was used [20]. The main advantage of this tool was the combination of three main positive selection sweep signatures presented as a μ value. This value described the effect of positive selection on genomic loci. Positive natural selection sweep signatures under analysis are (1) local reduction at the polymorphism level; (2) particular shift in the site frequency spectrum toward low- and high-frequency derived variants; (3) localised pattern of linkage disequilibrium (LD) levels, characterised by high LD on each side of a beneficial mutation and low LD between loci located on different sides of the beneficial allele. Existing similar software and traditional positive selection signature identification methods (e.g., F_ST_, Tajima D) identify these signatures only separately. Other RAiSD tool advantages include the negligible computing memory requirements and the processing of large amounts of data in a relatively short time. RAiSD tool was designed to detect positive selection signatures when the population of interest has been exposed to selective pressures over multiple generations. Thus, further results show positive selection signatures gained before the catastrophe. However, these genomic loci under positive selection may still affect the adaptation and health of LCWC. In this study, we were not looking for causality between the impact of the catastrophe (IR dose, psychological trauma, etc.) and the effect of positive selection after the disaster. Instead, we aimed to determine the positive selection signatures already present in the genomes before the disaster and their influence on the adaptation to the calamity and health in the cohort of LCWC. Two datasets of LCWC and LTU μ values, calculated from the WGS data, were compared. Analysis was performed for 22 autosomes. The effect of a positive natural selection was considered at μ > 0. The higher the value of μ, the stronger the impact of positive selection in the analysed genomic locus. Additionally, to identify the most significant loci under positive natural selection, it was assumed that the majority of the genome was neutral, i.e., not affected by positive selection. Loci were considered significant when they reached the top 5% of the highest μ estimates. This 5% value of the highest estimates corresponds to the *p*-value and was used as the significance threshold [20]. For further in silico biological function analysis of the identified top 5% significant loci under positive selection, we chose to only include loci with the highest value of μ in each autosome. This strategy was selected with the aim to analyse and cover all autosomes equally. The identification of loci in chromosomes 4 and 16 was performed manually using RAiSD Report files and Microsoft Office Excel software to filter out the top 5% significant loci under positive selection (due to the software specifics).

Identified loci were analysed using the UCSC genome browser [34]. Functional annotation was conducted using Ensembl [35], OMIM [36], GeneMANIA [37], PopHumanScan [38], PANTHER [39], Gene Ontology Browser [40], and Human DNA Repair Genes [41].

## 3. Results

**Figure 1:**
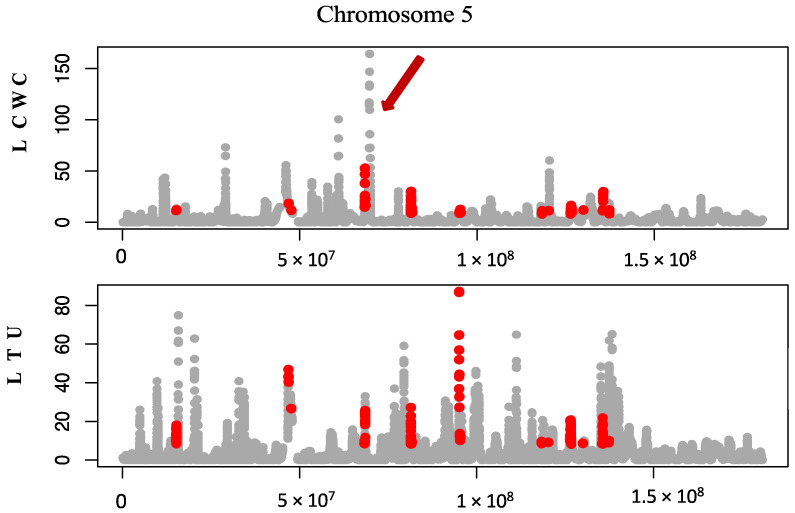
Analysis of the loci under positive selection on chromosome 5. The cohorts of the Lithuanian clean-up workers of the Chornobyl nuclear disaster (LCWC) and the general Lithuanian population (LTU) were compared. *X*-axis—genomic position; *Y* axis—positive selection signature μ value. The unique positive selection loci are marked grey, and the common ones are marked red. An arrow depicts the positive selection locus unique to LCWC, which has the highest μ value. The results of the analysis of positive natural selection for other chromosomes are provided in Supplementary Figure S1.

Following the comparison of genomic loci under significant positive selection, 22 loci (one in each autosome) unique to the LCWC cohort were identified. In the genomes of LCWC, the autosomal loci with the highest positive selection signature values were determined for chromosomes 5 (μ = 164.2 [Figure 1]), 11 (μ = 114), and 12 (μ = 161.1) (Table 1, Supplementary Figure S1). Identified loci under positive natural selection in chromosomes 9, 13, 18, and 21 do not contain genes; for this reason, these regions were not analysed further in this study.

**Figure 2:**
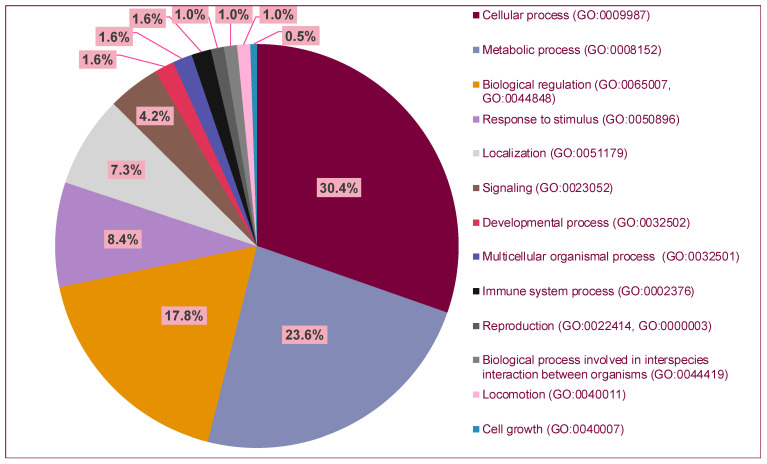
The biological role of the genes under positive natural selection. Processes in the legend are listed in descending order by the number of genes involved. Analysis is based on the PANTHER classification system [39] and the Gene Ontology Browser [40].

In total, 123 genes under positive selection in the LCWC group were identified. Analysis results of the biological function of the identified genes under the positive selection based on the PANTHER classification system [39] and the Gene Ontology Browser [40] are shown in Figure 2.

## 4. Discussion

Biological role analysis of the identified loci shows that most of the genes (71.8%) are involved in three main groups of biological processes: cellular, metabolic, and biological regulation (Figure 2). The type of cellular processes includes processes that are important for cell viability, such as cell division, cell cycle regulation, and cell death. The category of metabolic processes includes genes involved in cellular chemical reactions. Macromolecular processes such as DNA damage repair or DNA replication also fall into this category. The category of biological regulation involves a broad spectrum of processes, encompassing not only the regulation of biological processes in specific tissues and organs but also the regulation at the molecular level, such as the maintenance of DNA stability or transcription. Some identified genes have multiple biological roles and therefore fall into several categories. In this analysis, positive natural selection in LCWC genomes is shown to affect genomic loci that are involved in processes essential for cell survival (cell ageing, cell cycle regulation, apoptosis, and DNA damage repair).

The Lithuanian clean-up workers of the Chornobyl nuclear disaster experienced higher than usual doses of IR and encountered extreme and stressful situations. These situations led to mental disorders among Chornobyl power plant disaster clean-up workers from Lithuania and other affected countries [23,42,43]. The LCWC of this study have survived and adapted to the consequences of the disaster, therefore, the genes involved in DNA damage repair and the regulation of the cell cycle are of paramount importance for their adaptation and health. The identified genes involved in the aforementioned processes are: *CDKN2C, ZNF827, CENPH, CCNB1, CDK7, TAF9, RAD17, GTF2H2C, PTP4A1, WDR11, ALKBH2, UNG, ARID3B, COMMD, NEIL1, RFWD3, MACROD2, MAPK1, PPM1F,* and *TOP3B* (Table 2). These genes are also subject to positive natural selection and may form the unique adaptive properties of LCWC.

The PopHumanScan database [38], which provides systematic information on known loci under positive selection in different populations, has been used to further analyse the effects of the identified LCWC loci. For five genes (*WDR11, NEIL1, RFWD3, MACROD2,* and *MAPK1*; Table 2) involved in the DNA damage repair and cell cycle, the effect of positive natural selection was also observed in other populations. This demonstrates the significance of these regions for human adaptation processes in general, regardless of population. Otherwise, the influence of positive natural selection for other analysed genes (Table 2) in different world populations [38] and LTU was not determined during this study or was insignificant. This supports the idea that LCWC could be in part characterised by a specific genetic variation, which explains their survival and adaptation to the lifelong consequences of the Chornobyl nuclear disaster clean-up.

Aside from the DNA damage repair or cell cycle, we have identified more genes under positive natural selection that may affect the adaptive properties of LCWC. The *DAO* gene is in chromosome 12 and encodes a peroxisomal enzyme required for neuronal differentiation and dopamine synthesis [44]. It is associated with ageing and may have a neuroprotective effect [45]. Interestingly, the *DAO* gene is also associated with the oxidative stress response [46], which is one of the mechanisms experienced after receiving high doses of IR [47,48], and therefore may play a role in the response to oxidative stress and better adaptiveness in the LCWC cohort. In the same genomic region is the *PPTC7* gene, which is associated with resistance to environmental chemical toxins [49]. In the South Asian (Indian) population, this gene locus was found to be under positive natural selection as well (Fu and Li D score −6.825, *p* < 0.05) [38]. India is the third country in the world in terms of pollution [50], so it is natural for the population to adapt to the existing harmful environmental conditions. This correspondence of toxic conditions-experienced cohorts showing a positive natural selection on the *PPTC7* gene suggests the potential influence of this locus on human adaptation to toxicity.

Moreover, many of the identified genes affected by positive natural selection are associated with cancer processes, although not clear in what way. As per analysed questionnaire information, studied LCWC men exhibited fewer cancer cases than could be expected in the general population and, at the same time, experienced larger than usual doses of IR, which is known for its carcinogenic effects. Two out of 40 LCWC (5%) had malignant tumours (rectum or bronchus and lung). In the same age group of men in the Lithuanian population, the incidence of cancer was 8.2% in 2021 [51]. The fact that cancer-associated genome regions are under positive selection and the incidence of cancer may be lower in the studied group of LCWC suggests a possible protective function/activity of these loci against cancer and involvement in adaptiveness that determine LCWC survival.

## 5. Conclusions

The combination of new positive natural selection signatures and functional in silico analysis methods allowed us to identify genomic loci that are potentially important for the adaptation and survival of the LCWC. We demonstrate that DNA damage repair and cell cycle-involved coding regions are significant for human adaptation in general, regardless of population. We also show that the *DAO* gene could be an essential protective agent of oxidative stress response mechanisms to IR as well as *PPTC7*—to pollution. We reveal an existing paradox in cancer morbidity among the LCWC survivors’ group, which could be partially explained by the protective activity of cancer-related genes. Our results justify our initial hypothesis, that LCWC genomes are unique and could contain genomic factors accounting for protective health-related effects and adaptiveness. However, we would like to outline that our results may have potential limitations arising from the possible differences among the sample and control groups—age, lifestyle, morbidities, etc. Therefore, our results should be interpreted more as qualitative rather than quantitative.

Characterising positive selection signature loci in cohorts such as ours might set a good precedent for studies identifying genomic loci relevant to survival, longevity, and adaptiveness, and provide a better understanding of ongoing microevolutionary processes, multifactorial traits, and diseases. These loci could be used as potential targets for gene therapy to create new disease diagnostics, prevention, and treatment strategies, thus moving towards personalised medicine.

## Figures and Tables

**Table 1:** Autosomal regions under the positive natural selection.

Chromosome	Start Genomic Position	Length of the Region	Highest μ Value	Genes
1	50024718	1,562,262	49.96	*AGBL4, ELAVL4, DMRTA2, FAF1, CDKN2C*
2	189198631	532,713	86.85	*GULP1, DIRC1*
3	68678446	312,015	77.44	*TAFA4*
4	146494457	860,240	72.27	*MMAA, C4orf51, ZNF827, LSM6, SLC10A7*
5	68316218	1,615,807	164.20	*SLC30A5, CCNB1, CENPH, MRPS36, CDK7, CCDC125, AK6, TAF9, MARVELD2, OCLN, GTF2H2C, SERF1B, SMN2, NAIP*
6	64047472	568,457	56.06	*PTP4A1, PHF3, EYS*
7	151965978	167,002	28.44	*KMT2C*
8	104888906	2,138,519	36.35	*RIMS2, DCSTAMP, DPYS, LRP12, ZFPM2*
9	17876558	452,637	54.64	*-*
10	122646955	434,876	41.73	*WDR11*
11	101273279	466,877	114.00	*TRPC6*
12	109191005	2,131,765	161.10	*SSH1, DAO, USP30, ALKBH2, UNG, ACACB, MYO1H, KCTD10, MMAB, UBE3B, MVK, TRPV4, GIT2, GLTP, ANKRD13A, C12orf76, IFT81, ATP2A2, ANAPC7, GPN3, ARPC3, FAM216A, VPS29, PPTC7, RAD9B, TCTN1, HVCN1, PPP1CC, CCDC63*
13	82488223	980,137	30.54	*-*
14	73733462	133,836	61.43	*PAPLN, NUMB, AX747833*
15	74491439	1,491,519	59.46	*CCDC33, CYP11A1, SEMA7A, UBL7, ARID3B, CLK3, EDC3, CYP1A1, CSK, ULK3, SCAMP2, FAM219B, MPI, COX5A, RPP25, SCAMP5, PPCDC, C15orf39, GOLGA6C, MAN2C1, COMMD4, NEIL1, SIN3A, PTPN9, SNUPN*
16	74347008	448,159	29.69	*NPIPB15,* *CLEC18B,* *GLG1, RFWD3, MLKL, FA2H*
17	49795141	746,430	27.98	*CA10*
18	68939522	52,295	29.70	*-*
19	22058047	948,331	72.65	*ZNF208, ZNF257, ZNF676, ZNF729, ZNF98, ZNF492, ZNF99*
20	14021037	45,777	22.27	*MACROD2*
21	23193282	405,462	41.25	*-*
22	21806118	847,114	45.96	*TMEM191C, PI4KAP2, UBE2L3, CCDC116, SDF2L1, PPIL2, YPEL1, MAPK1, PPM1F, TOP3B*

**Table 2:** List of the identified genes under positive natural selection involved in the DNA damage repair and cell cycle, which may contribute to the survival and well-being of LCWC. Function or impact on the phenotype was determined from data provided in the UCSC genome browser, OMIM, Human DNA Repair Genes databases, and analysis of scientific publications.

Chromosome	Gene	Function and (or) Impact on Phenotype
1	*CDKN2C*	Cell cycle G1 control, tumour suppression, cancer, endocrine dysplasia
4	*ZNF827*	Reparation of telomeres, associated with alcohol-related liver disease
5	*CENPH*	Forms centromere-kinetochore complex, cancer
5	*CCNB1*	Cell cycle G2-M control, cancer
5	*CDK7*	Cell cycle and transcription, nucleotide excision repair
5	*TAF9*	Cell cycle, apoptosis, DNA transcription
5	*RAD17*	DNA lesions-depended recombination, DNA damage sensor.
5	*GTF2H2C*	DNA damage repair
6	*PTP4A1*	Cell cycle G1-S control, cancer, systemic sclerosis, alcohol dependence
10	*WDR11*	Cell cycle progression, apoptosis, signalling, transcription regulation, associated with obesity, intellectual disability, cancer, heart anomalies
12	*ALKBH2*	Direct reversal of DNA damage
12	*UNG*	Reparation of telomeres, base excision repair
15	*ARID3B*	Cell cycle control, transcription regulation, regulation of chromatin structure
15	*COMMD4*	Repair of double-stranded DNA breaks
15	*NEIL1*	Base excision repair
16	*RFWD3*	DNA lesions repair in G1 phase of the cell cycle
20	*MACROD2*	Potential function in DNA repair, associated with cancer
22	*MAPK1*	Proliferation, differentiation, transcription regulation
22	*PPM1F*	Regulation of cellular stress response
22	*TOP3B*	Genome stability, DNA recombination, ageing

## Data Availability

All data generated or analysed during this study are included in this published article and its Supplementary information files. Additional data may be available upon request.

## Supplementary Materials

The following supporting information can be downloaded at: https://www.mdpi.com/article/10.3390/cimb45040195/s1, Figure S1: Analysis of the positive selection signatures on autosomes in the genomes of Lithuanian clean-up workers of Chornobyl nuclear disaster (LCWC).
