# Supplementary material for: Identifying Genomic Signatures of Positive Selection to Predict Protective Genomic Loci in the Cohort of Lithuanian Clean-Up Workers of the Chornobyl Nuclear Disaster
Source: Curr Issues Mol Biol. 2023 Apr 3;45(4):2972–83. doi: 10.3390/cimb45040195 (PMC10137185; doi:10.3390/cimb45040195)

Supplementary Figure S1. Analysis of the positive selection signatures on autosomes in the genomes of Lithuanian clean-up workers of Chernobyl nuclear disaster (LCWC). Lithuanian clean-up workers of Chernobyl nuclear disaster and the general Lithuanian population (LTU) groups were compared. X-axis – genomic position, Y-axis – μ value. Values under positive natural selection, unique to the LCWC study group and with the highest μ values are marked with arrows. Red-marked genomic loci depicts identical genomic loci under positive selection between LCWC and LTU study groups. Gray-marked genomic loci regions depicts non-identical (unique) genomic loci under positive selection. Due to an artificially increased centromeric signal in chromosomes 4 and 16, schematic representation was not possible and identification of unique genomic loci was performed manually using *RAiSD* *Report* files.


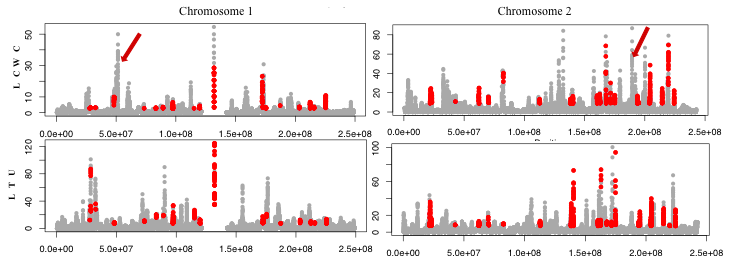


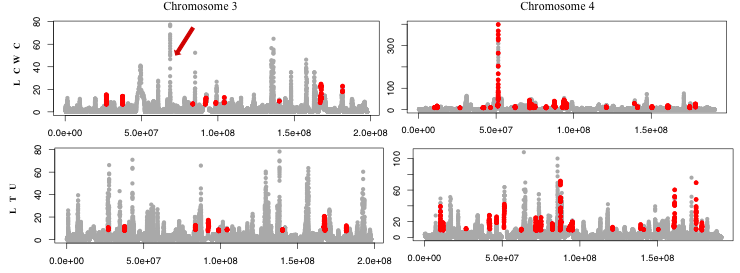


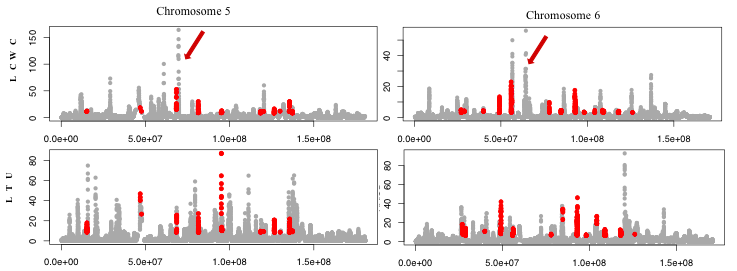


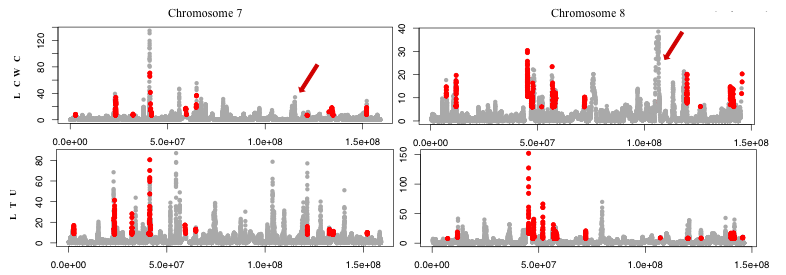


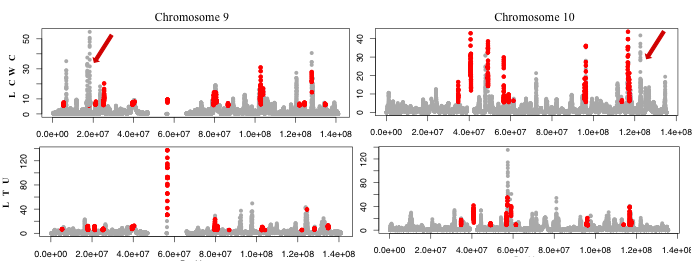


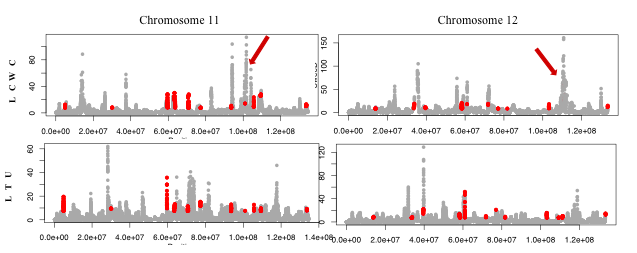


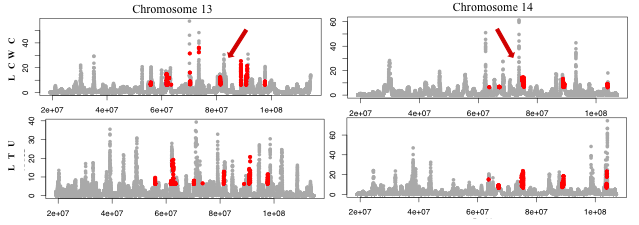


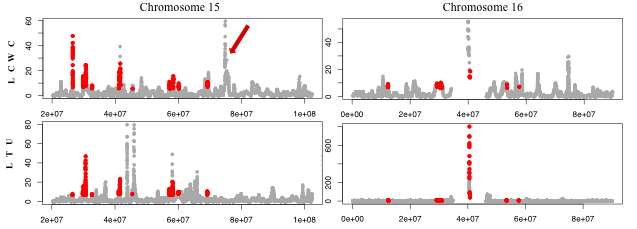

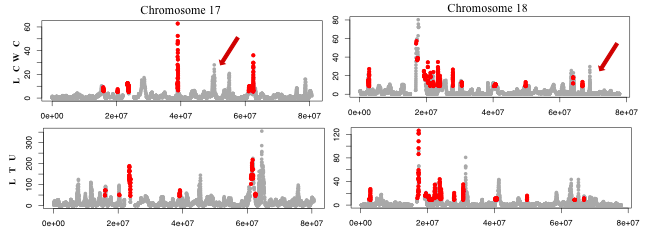


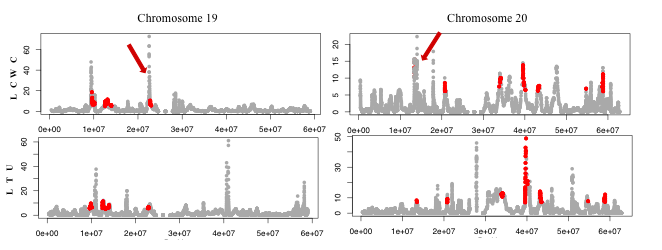


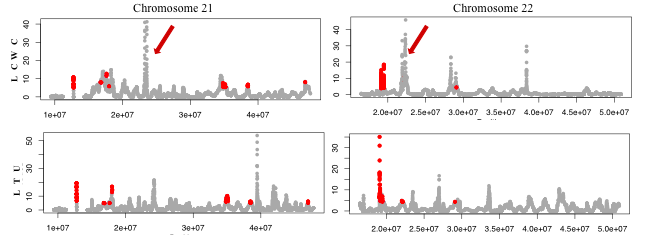

Supplement: Supplementary file 1 [file cimb-45-00195-s001.zip › cimb-2203014-supplementary.docx]
