# Supplementary material for: Identifying Genomic Signatures of Positive Selection to Predict Protective Genomic Loci in the Cohort of Lithuanian Clean-Up Workers of the Chornobyl Nuclear Disaster
Source: Curr Issues Mol Biol. 2023 Apr 3;45(4):2972–83. doi: 10.3390/cimb45040195 (PMC10137185; doi:10.3390/cimb45040195)
Supplement: Supplementary file 1 [file cimb-45-00195-s001.zip › cimb-2203014-supplementary.pdf]

Supplementary Figure S1. Analysis of the positive selection signatures on autosomes in the genomes of Lithuanian clean-up workers of Chernobyl nuclear disaster (LCWC). Lithuanian clean-up workers of Chernobyl nuclear disaster and the general Lithuanian population (LTU) groups were compared. X-axis – genomic position, Y-axis – μ value. Values under positive natural selection, unique to the LCWC study group and with the highest μ values are marked with arrows. Red-marked genomic loci depicts identical genomic loci under positive selection between LCWC and LTU study groups. Gray-marked genomic loci regions depicts non-identical (unique) genomic loci under positive selection. Due to an artificially increased centromeric signal in chromosomes 4 and 16, schematic representation was not possible and identification of unique genomic loci was performed manually using *RAiSD Report* files.

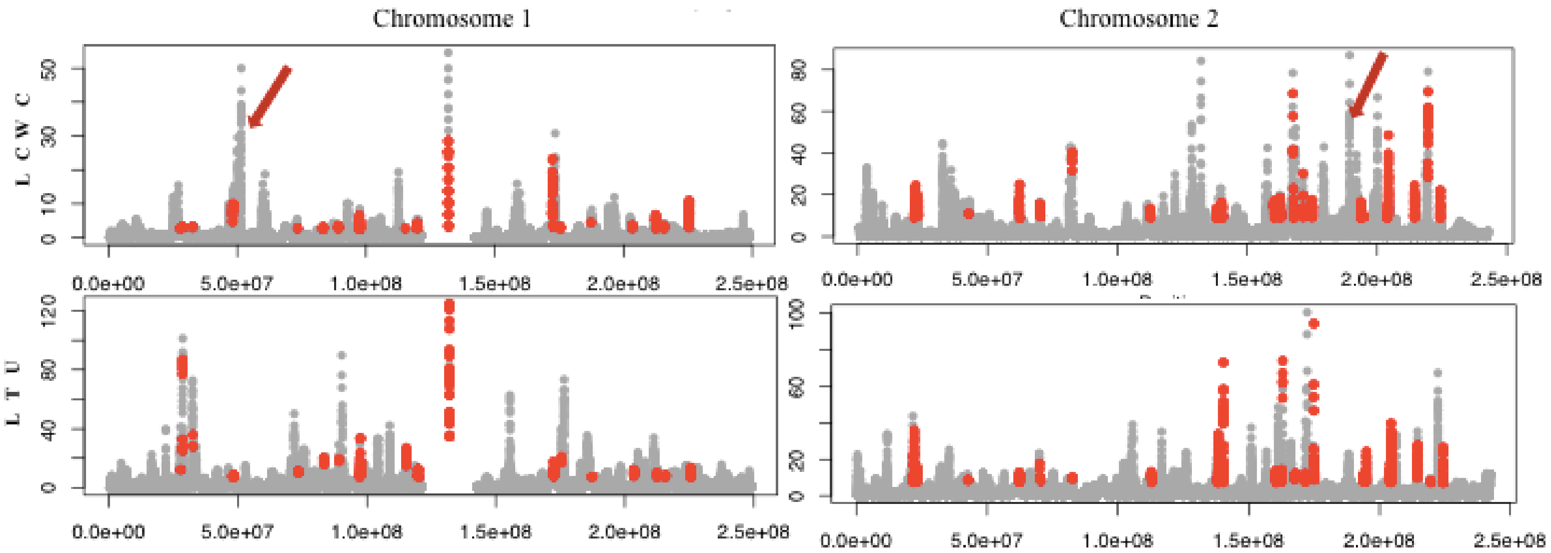

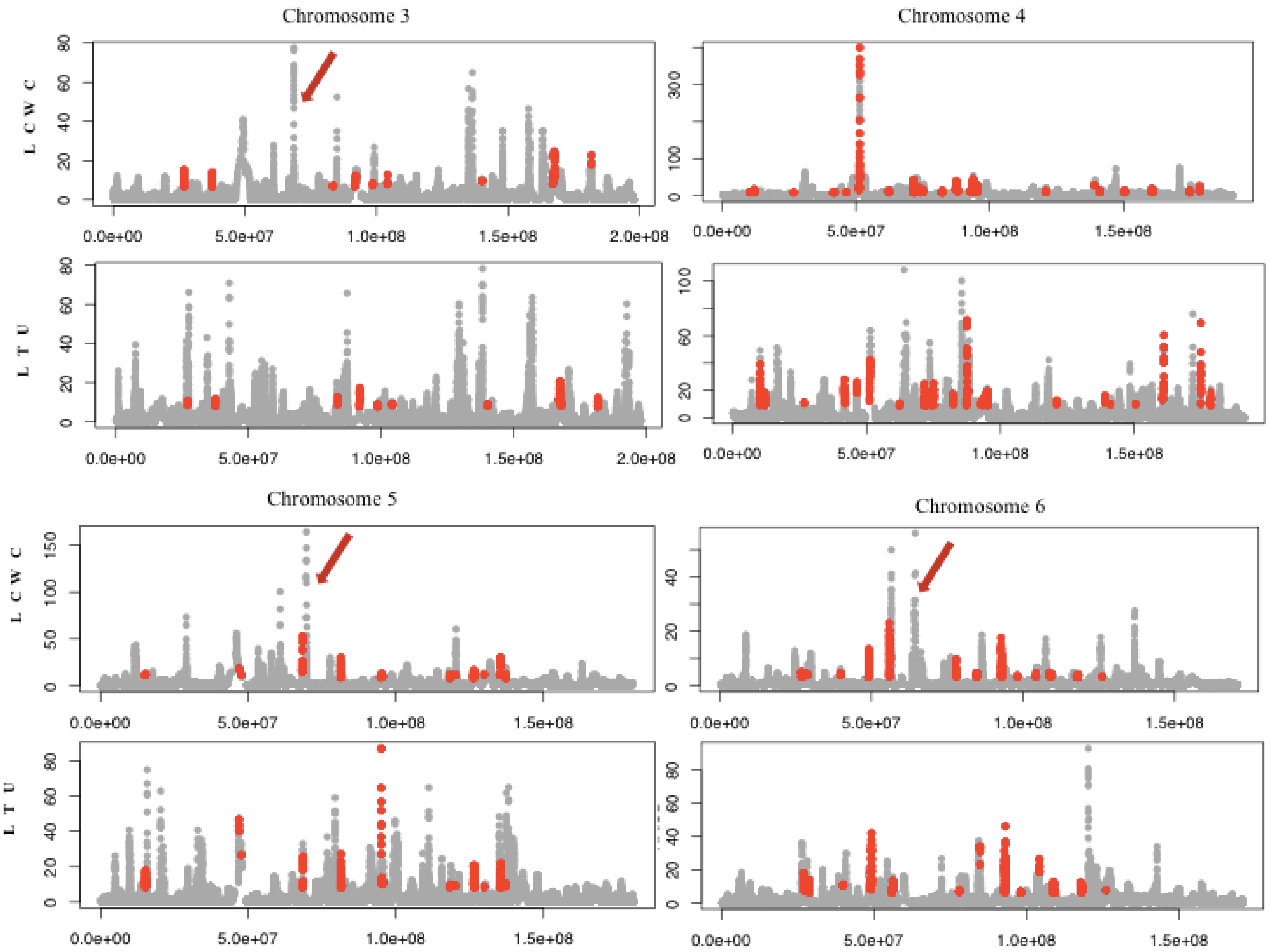

Chromosome 3
Chromosome 4
Chromosome 5
Chromosome 6
L C W C
L T U
L C W C
L T U
0.0e+00
5.0e+07
1.0e+08
1.5e+08
2.0e+08

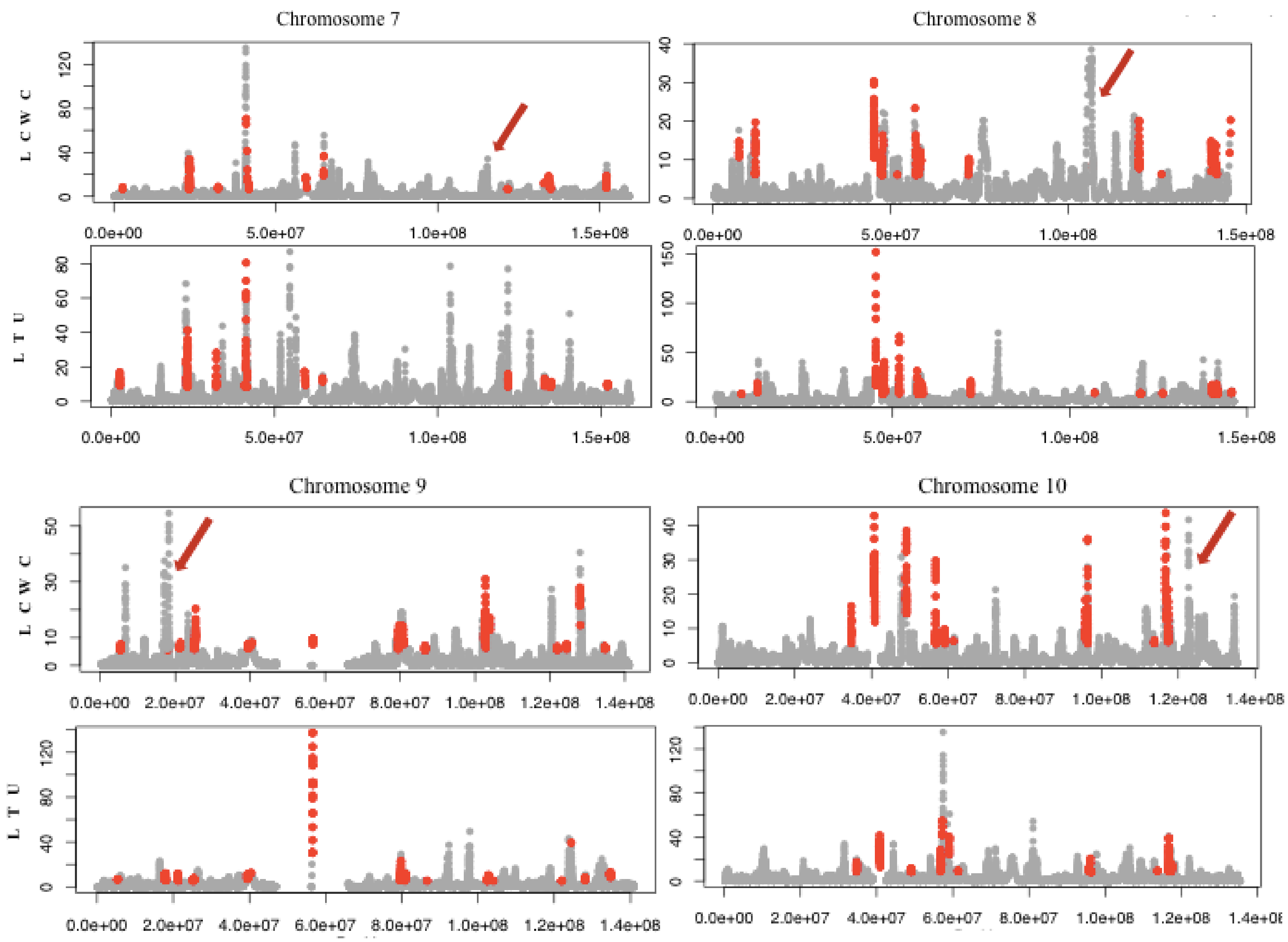

Chromosome 7
Chromosome 8
Chromosome 9
Chromosome 10
LCWC
LTU
0.0e+00
5.0e+07
1.0e+08
1.5e+08
2.0e+07
4.0e+07
6.0e+07
8.0e+07
1.2e+08
1.4e+08

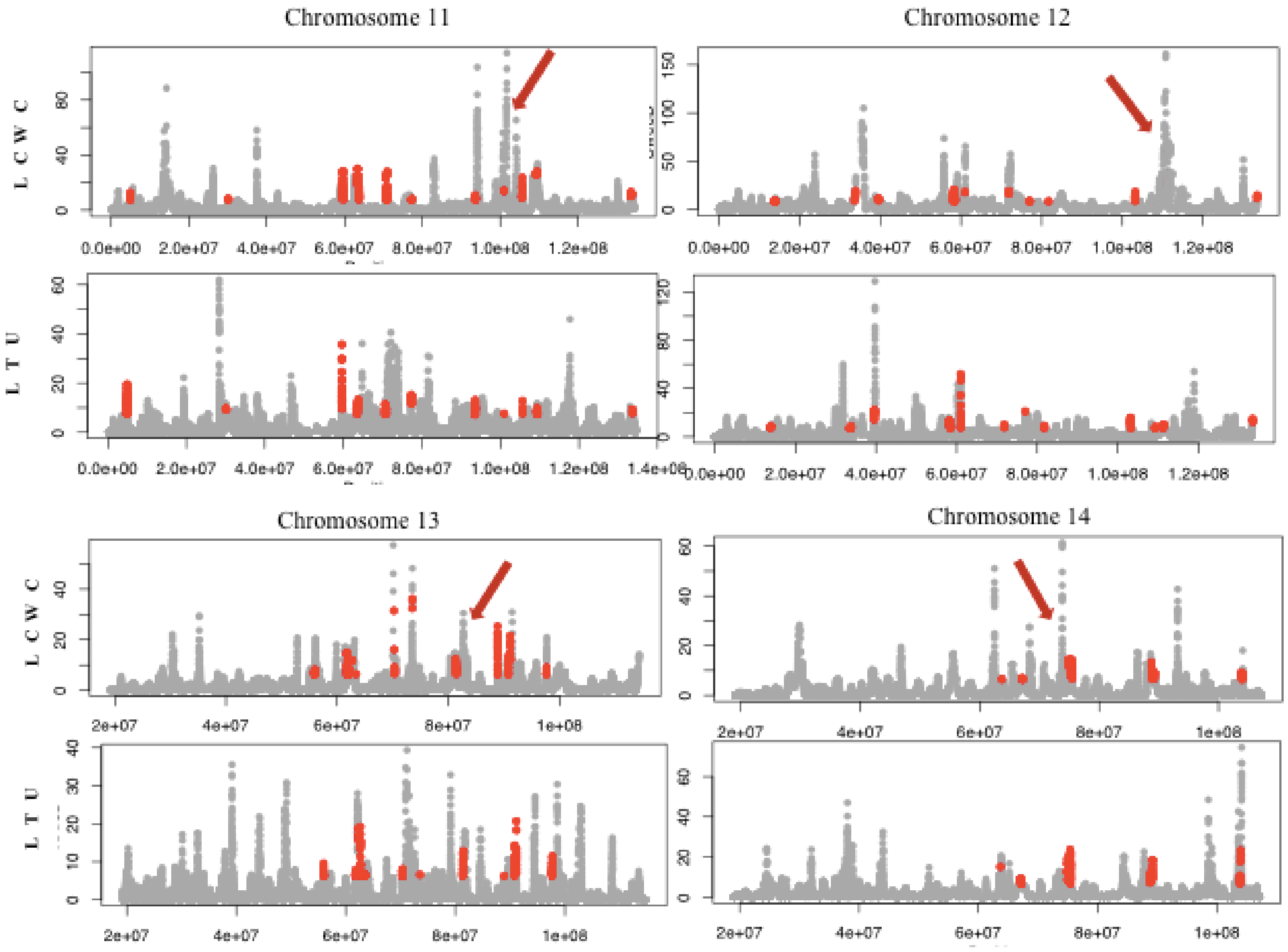

Chromosome 11
Chromosome 12
Chromosome 13
Chromosome 14
L C W C
L T U
0.0e+00
2.0e+07
4.0e+07
6.0e+07
8.0e+07
1.0e+08
1.2e+08
1.4e+08
2e+07
4e+07
6e+07
8e+07
1e+08

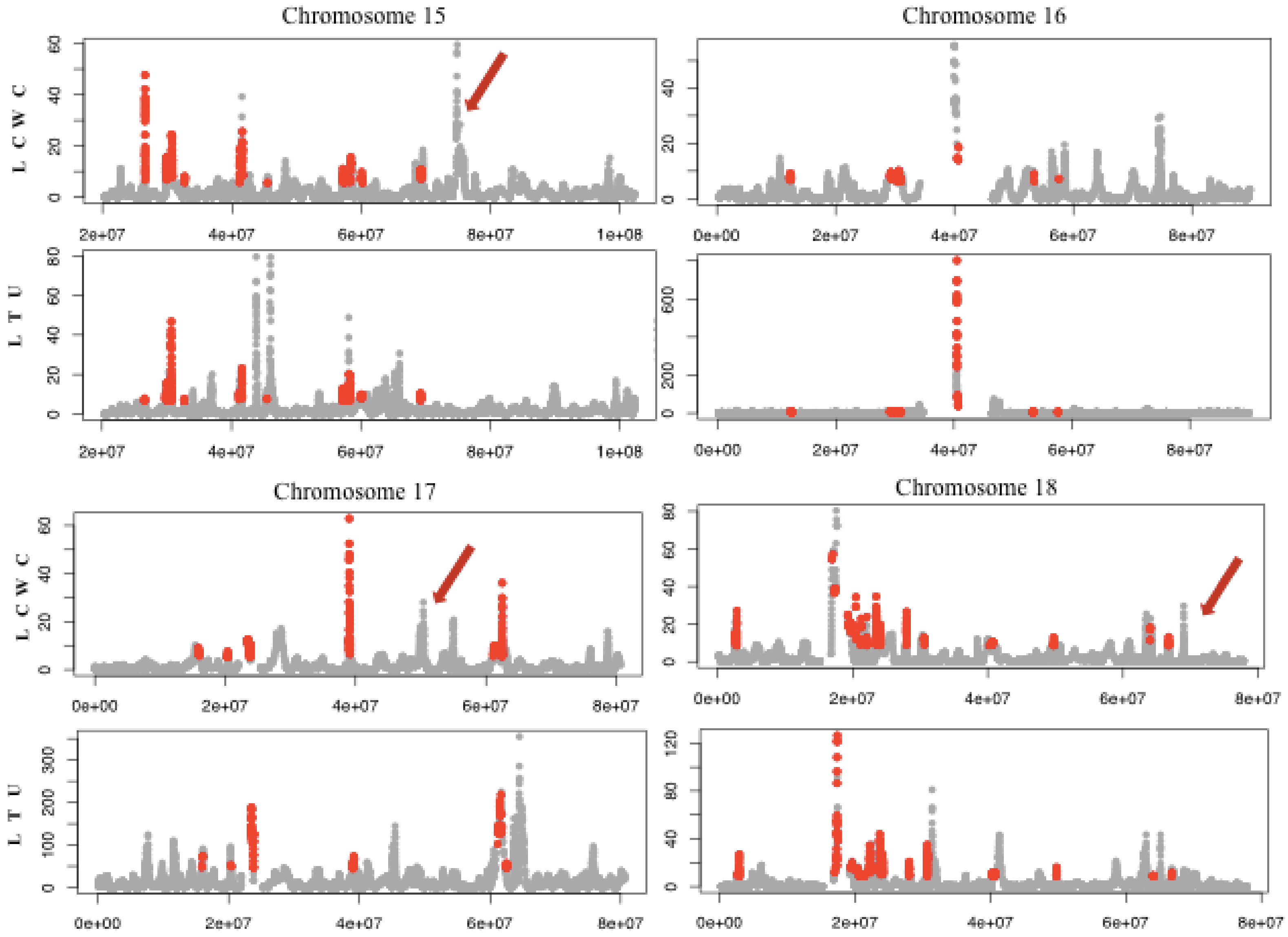

Chromosome 15
Chromosome 16
Chromosome 17
Chromosome 18
L C W C
L T U

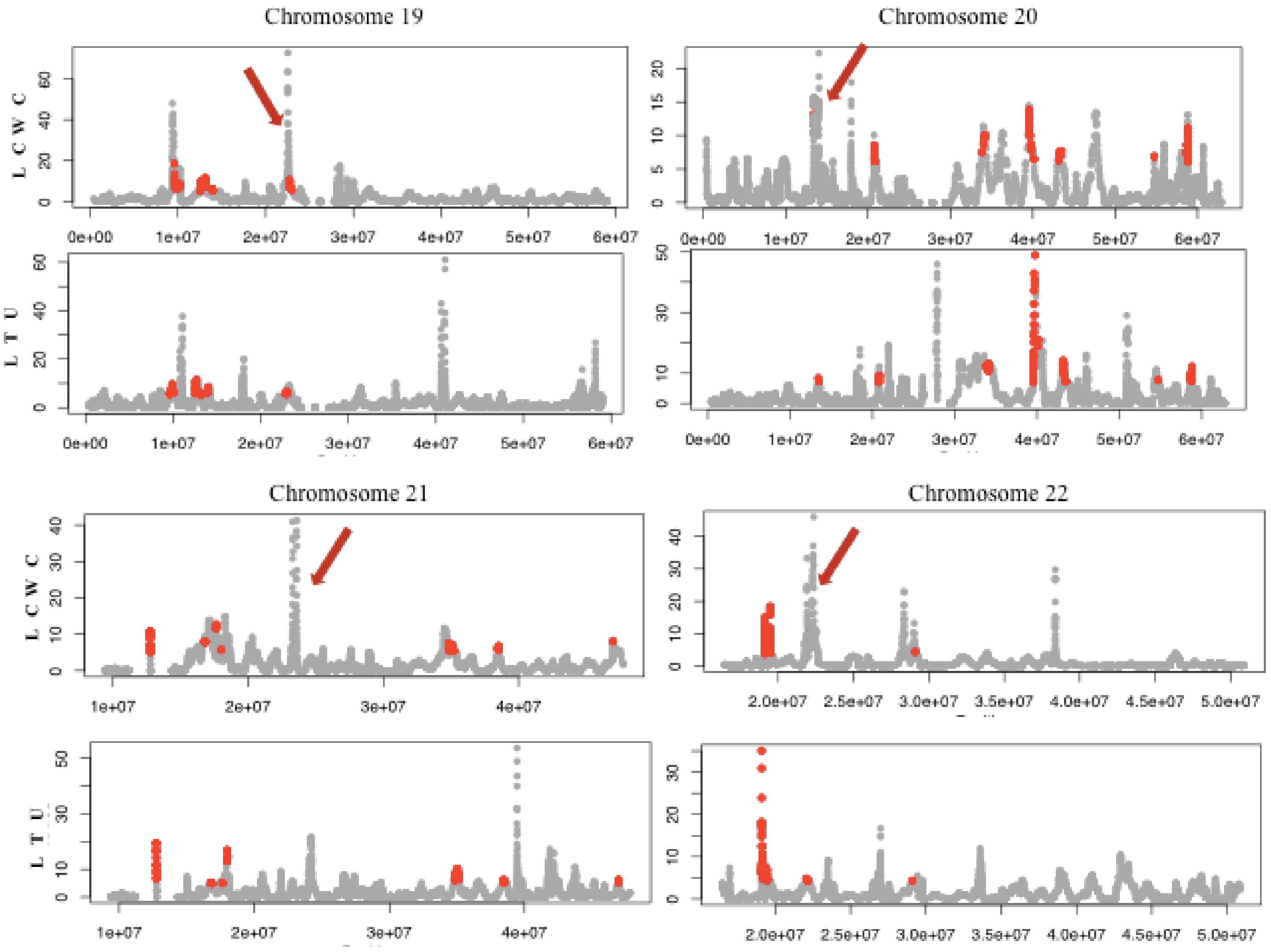
Chromosome 19
Chromosome 20
Chromosome 21
Chromosome 22
L C W C
L T U
